# Hemophagocytic lymphohistiocytosis: epidemiological, clinical and biological profile

**DOI:** 10.3906/sag-1812-65

**Published:** 2019-10-24

**Authors:** Hanane ZAHIR, Jihane BELKHIR, Hanane MOUHIB, Mustapha AIT AMEUR, Mohammed CHAKOUR

**Affiliations:** 1 Laboratory of Hematology, Avicenna Hospital of Marrakesh, Faculty of Medicine and Pharmacy, Cadi Ayyad University, Marrakesh Morocco

**Keywords:** Cytopenia, hemophagocytic lymphohistiocytosis, inflammation, hemophagocytic syndrome, myelogram

## Abstract

**Background/aim:**

Hemophagocytic lymphohistiocytosis (HLH) is a clinical, biological, and pathological entity that is rare but has certain morbidity that may be life-threatening. This work aims to establish a focus on the hemophagocytic lymphohistiocytosis and analyze different aspects of diagnosis while emphasizing the biological data.

**Materials and methods:**

We report the results of a retrospective study conducted in the hematology department of Avicenna Hospital in Marrakesh. Thirty-one patients with hemophagocytic lymphohistiocytosis were enrolled.

**Results:**

The clinical presentation was dominated by fever and deterioration of the general state for almost all our patients. Splenomegaly was objectified in 90% of the patients. Hepatomegaly, lymphadenopathy, and hemorrhagic manifestations were observed in almost 50% of the patients. Biological assessments revealed bi- or pancytopenia in 96% of the patients, and coagulation disorders in 51% of the patients. On the other hand, hyperferritinemia was found in 84% of the patients, and hepatic cytolysis and hypertriglyceridemia in half of the patients. Hemophagocytosis was observed in all bone marrow samples taken from our patients. Concerning the evolution of patients, in 38.5% of the patients, the evolution was favorable with regression of clinical and biological signs. Twenty six percent of the patients had died, mainly from multiple organ failure and disseminated intravascular coagulation.

**Conclusion:**

HLH is a diverse condition with many causes and is likely to be under-recognized, which contributes to its high morbidity and mortality. Clinicians need to be able to recognize the signs and symptoms commonly seen in HLH and actively pursue this diagnosis in the cases of undiagnosed febrile illness with multiorgan dysfunction. Early recognition is crucial for any reasonable attempt at curative therapy to be made.

## 1. Introduction

Hemophagocytic lymphohistiocytosis (HLH) is a complex clinical–biological association resulting from the inappropriate activation and proliferation of cells from the lymphohistiocyte lineage. It is still unknown and its diagnosis is frequently delayed in some patients given the atypical and polymorphous presentation of its manifestations.

It is a rare disorder, affecting both the adult and the child whose etiologies and pathophysiological mechanisms are multiple, which accounts for the diagnostic and therapeutic difficulties. This work aims to establish a focus on the HLH through these cases and to analyze the different aspects of the diagnosis while emphasizing the biological data.

## 2. Patients and methods

This is a descriptive and analytical retrospective study over a period of seven years from April 2011 to March 2018, including about 31 cases of HLH, using the data collected in the hematology laboratory of the Avicenna Hospital of Marrakesh.

Thus, the diagnosis of HLH was retained in the presence of clinical and biological signs, according to the diagnostic criteria of Henter et al. 2007 [1]: fever, splenomegaly, cytopenia (hemoglobin (Hb) <90 g/L, platelets <100 × 109/L, neutrophils (PNN) <1 × 109 /L), hypertriglyceridemia (>3 mmol/L) and/or hypofibrinemia (<1.5 g/L), hemophagocytosis (marrow), ferritin >500 mg/L, soluble CD25 >2400 UI/mL, and natural killer activity zero or lower.

The blood count was determined on a calibrated and controlled “Sysmex® XT 4000” automated system, supplemented by a May-Grünwald-Giemsa stained blood smear, and then observed under a microscope. The medullary punctures were performed on the sternum next to the second intercostal space for adults, and the anterior superior iliac spines for children. The smears were spread on glass slides (medullary smear), then observed under a microscope after May-Grünwald- Giemsa staining using the manual method. For each patient, at least two independent readings of blood and bone marrow smears were performed and validated by the cytologists. The hemostasis assessment was performed on the “Stago® STA Compact” automaton and the biochemical report on the “Cobas® 6000” automaton.

The data was collected from the medical records of the patients. The exploitation of the data was carried out by using the Excel© software, and the statistical analysis using the software SPSS version 10 for Windows. The data analyzed in this study are the epidemiological, clinical, and biological data of this syndrome.

## 3. Results

The average age of the patients was 35 years with extremes ranging from 12 to 57 years old. Of these patients, 61% were males, which gives a sex ratio of 1.58. Five patients had an underlying etiology, the first patient was followed for Hodgkin lymphoma, the second one had systemic lupus erythematosus, the third had severe combined immunodeficiency, the fourth had juvenile idiopathic arthritis, and the fifth had a known asthma and noninsulindependent diabetese. The delay in management was variable with an average of 28 days, and extremes ranging from 10 days to 2 months.

The clinical presentation of the patients was dominated by fever and deterioration of the general state. Ninety percent of the patients had splenomegaly. Hepatomegaly, lymphadenopathy, and hemorrhagic manifestations were present in almost 50% of the patients. Other clinical signs have been found, including cutaneous and pulmonary manifestations. The clinical presentation of our patients is shown in Table 1.

**Table 1:** Clinical presentation of patients.

Symptom	Frequency
Fever	100%
Deterioration of the general state	93.5%
Splenomegaly	90.3%
Hemorrhagic manifestations	51.6%
Hepatomegaly and/or lymphadenopathy	48.4%
Skin damage	9.7%
Pulmonary impairment	3.2%

The biological presentation of our patients was dominated by abnormalities of the hemogram; cytopenia was found in all patients, in the form of pancytopenia in 67.7% of the patients and bicytopenia in 29% of the patients. Thrombocytopenia, less than 100 × 109/L, was the most frequently observed cytopenia (96.8% of the patients). Anemia (Hb < 90 g/dL) was indeed observed in 90.3% of the patients, it was normochromic normocytic in 83.9% and normochromic microcytic in 6.4% of the patients. Neutropenia was observed in 77.4% of patients, leukocytosis in PNN in 6.4% of the patients. In the myelogram, the marrow was rich in 71% of the patients. All patients had signs of macrophage activation with images of haemophagocytosis (100%), the percentage of activated macrophages exceeded 3% in 83% of the patients. Medullary smear abnormalities were documented in all our patients with varying frequencies, erythroblastosis in 25.8% of the patients, monocytosis in 12.9%, and hyperplasia of the lymphoid lineage in one patient.

A disturbance of the haemostasis assessment was observed in 51.6% of the patients, with an extension of the Quick Time (TQ) in 51% of the patients and the Partial Thromboplastin time and Activator (TCA) in 48% of the patients.

Regarding the biochemical assessment, the increase in lactate dehydrogenases (LDH) was almost constant (87%), as was the hyperferritinemia (84%). Triglycerides was high in 51.6% of the patients. Hepatic cytolysis was also present in almost half of the patients. An inflammatory syndrome marked by increased sedimentation rate and / or reactive protein C (CRP) was observed in 80% of the patients. The main abnormalities of the biological assessment found are illustrated in Table 2.

**Table 2:** The main biological anomalies observed in our series.

Biological anomaly	Percentage
Anemia (Hb < 90 g/L)	90.3%
Thrombocytopenia (PQ < 100 × 109/L)	96.8%
Neutropenia (PNN < 1 × 109/L)	77.4%
TQ lengthened ( ratio > 1.3)	51.6%
TCA lengthened ( ratio > 1.3)	48.4%
Elevated transaminases (ALAT/ASAT > 60 UI/L)	51.6%
GGT and / or PAL high (GGT > 55 UI/L ; PAL > 200 UI/L)	32.2%
High LDH (LDH > 220 UI/L)	87.1%
Hypertriglyceridemia (TG > 3 mmol/L)	51.6%
Hyperferritinemia ( > 500 mg/L)	83.9%
Hyponatremia (Na+ < 128 mmol/L)	19.3%
High blood sugar (Gly > 7 mmol/L)	9.7%
Sedimentation rate > 25 mm and / or CRP > 6 mg/L	80.6%

Concerning the evolution of patients, in 38,5% of patients, the evolution was favorable with regression of clinical and biological signs. Twenty six percent of patients died, mainly for multiple organs failure and disseminated intravascular coagulation. Follow up data was lost for 11 of the patients (35,48%).

## 4. Discussion

HLH is a rare, serious clinical, biological, and histological entity characterized by excessive activation of macrophages and T cells leading to a hyperinflammatory state. It is distinguished in primitive (related to a congenital immunodeficiency) and secondary (related to infections, neoplastic and autoimmune diseases).

The diagnostic criteria for HLH are those defined by Henter et al. in 2007 [1]. These criteria include on the one hand biological data: hematological and biochemical, and on the other hand genetic data. The diagnosis of HLH has evolved with the introduction, in particular, of new criteria directly related to the physiopathology, namely the increase of the soluble receptor levels of interleukin-2 (IL-2), sCD25, reflection of the hyperactivation of the immunological system, and decreased cytotoxicity functions of innate immunity cells called Natural killer. However we did not undertake within this study any genetic tests nor soluble CD25 and natural killer cell activity.

Clinically, HLH is characterized by a set of important general signs, organomegaly, and lymphadenopathy with neurological, digestive, pulmonary signs indicating multiorgan involvement. Its clinico-biological signs are not very specific but their association should suggest a diagnosis. The fever is often high. It is caused by the high level of proinflammatory cytokines such as tumor necrosis factor alpha (TNF-α), Macrophage Inflammatory Protein 1 alpha (MIP-1α), interferon gamma (IFN-γ), and IL-6 in particular [2,3]. In our series, all patients had a fever, which is consistent with the literature data [4–6]. The enlargement of the lymphoid organs can affect the lymph nodes, the liver, and the spleen. This organomegaly corresponds to an infiltration by lymphocytes and macrophages. Splenomegaly was present in 90% of the patients in our series. On the other hand, hepatomegaly and lymphadenopathies were found in almost half of our patients, which partly coincides with the results of Rivière et al.’s study [7].

In terms of biological abnormalities, hematological involvement was by far the most common. The cardinal sign of this attack is the presence of cytopenias secondary to phagocytosis of the hematopoietic elements [8]. Cytopenias are almost constant in the different series studied, in 89.4% of the patients in Larroche’s study [9] and in 99% of the patients in Li et al.’s study [10]. In agreement with the literature data, the biological sign found in the foreground of our study was cytopenias (100%); pancytopenia and bicytopenia were found in 67.7% and 29% of the patients, respectively. The mechanism of these cytopenias is both central and peripheral, explaining the little or no regenerative nature of anemia despite its hemolytic component. Hemophagocytosis plays a quantitative role that may not be important in these cytopenias which are more related to the secretion of TNF-α as well as IFN-γ [11].

Moreover, anemia has been reported in 90.3% of our observations, which is consistent with the results of several series of the literature [4,12]. It is found in 80% to 100% of the patients, it is often normocytic, normochromic, and aregenerative [6]. Thrombocytopenia is almost constant, found in more than 86% of the patients, often less than 100 × 109/L [6,13]. Thrombocytopenia was observed in 96.8% of our patients. Its mechanism is central, but also sometimes peripheral, notably through disseminated intravascular coagulation, which complicates transfusion management [6]. Leukopenia, appearing in 60% of the patients, is rather late. It is marked by lymphopenia and sometimes by profound neutropenia [6]. It was revealed in 77.4% of the patients in our series.

In myelogram, the typical cytological appearance of HLH is that of histiocytic and / or macrophage proliferation with images of hemophagocytosis [8]. This hemophagocytosis is important to the diagnosis of HLH, but it is not mandatory. Signs of hemophagocytosis are sought on the myelogram, but less frequently on lymph node biopsies or splenectomy specimens [11]. The increase of ferritin above 500 mg/L is part of HLH’s diagnostic criteria. It was observed in 83.9% of our patients. Hyperferritinemia is an indicator of macrophage activation and can reflect hyperproduction of TNF-α, indeed, most cases of hyperferritinemia are not caused by transfusional iron overload, but by the hyperproduction of TNF‐α. Moreover, although the serum ferritin cut‐off in the HLH‐2004 diagnostic criteria is ≥500 ng/mL, it often rises to more than several thousand ng/mL in patients with HLH [14].

Hypertriglyceridemia, which is not associated with increased cholesterol, seen in 51.6% of our patients, is part of the HLH picture and can itself be a cause of pancreatitis. This is due to the action of TNF‐α and Il-1 released by activated macrophages and inhibiting lipoprotein lipase. It is usual to observe liver abnormalities with elevated aminotransferases, alkaline phosphatases, bilirubin, and a moderate decrease in factor V, which indicates some degree of hepatic failure [11].

In conclusion, the HLH is a rapidly progressive, lifethreatening syndrome of excessive immune activation. It is associated with high mortality rate. Making the diagnosis of HLH could be quite challenging due to the broad range of presenting symptoms and their lack of specificity. Common findings include fever, hepatosplenomegaly, rash, lymphadenopathy, neurologic symptoms, cytopenias, high serum ferritin, and liver function abnormalities. Etiologically, secondary HLH, especially of infectious origins, remains the most frequent. Awareness of the clinical symptoms and of the diagnostic criteria of HLH is important to start life-saving therapy with immunosuppressive/immunomodulatory agents in time.
